# Ipsilateral pull-through technique using a handmade loop snare catheter for difficult port catheter removal

**DOI:** 10.1186/s42155-025-00646-8

**Published:** 2026-01-15

**Authors:** Tomomasa Matsuo, Atsushi Saiga, Rui Sato, Kazuhisa Asahara, Takeshi Aramaki

**Affiliations:** 1 Division of Interventional Radiology, Shizuoka Cancer Center, 1007 Shimonagakubo, Nagaizumi-cho, Sunto-gun, Shizuoka, 411-8777 Japan; 2 Division of Urology, Shizuoka Cancer Center, 1007 Shimonagakubo, Nagaizumi-cho, Sunto-gun, Shizuoka, 411-8777 Japan

**Keywords:** Totally implantable venous access devices, Ipsilateral pull-through technique, Handmade loop-snare catheter

## Abstract

**Purpose:**

Removal of an adhered indwelling catheter in a totally implantable venous access device (TIVAD) can occasionally be challenging, particularly after prolonged implantation. The purpose of this paper is to present a modified endovascular technique for difficult TIVAD removal and to highlight its clinical relevance in cases where the catheter is firmly adhered to the vessel wall, making standard removal methods challenging.

**Materials and methods:**

Between December 2015 and April 2025, a total of 3347 TIVADs were implanted, and 367 removal procedures were conducted. Among these, 355 (96.7%) catheters were successfully removed using the standard technique. Three (0.8%) were removed using the push-in techniques, and one (0.3%) was removed using the contralateral pull-through combined with the sheath-twist technique. In 8 (2.2%), the ipsilateral pull-through technique was required after failure of the initial approaches. The ipsilateral pull-through technique was performed using an introducer sheath and a handmade loop-snare constructed from a guidewire and a seeking catheter. Patient characteristics and procedural data were collected for analysis.

**Results:**

The ipsilateral pull-through technique was successfully used to remove difficult-to-remove TIVADs in 7 of 8 patients. In the remaining patient, the indwelling catheter was firmly adhered to the segment extending from the innominate vein to the superior vena cava, where antegrade flow was absent and numerous collateral vessels were present. Although the occlusion was successfully crossed, catheter removal was aborted due to severe pain and concerns about potential superior vena cava rupture. One procedure-related complication was observed: catheter fracture on follow-up computed tomography in one of the seven successful cases. No other complications were observed. The subclavian vein, innominate vein, and superior vena cava were patent on follow-up computed tomography performed for cancer evaluation. The median indwelling duration in this cohort was 2473 days (interquartile range [IQR], 2017–3002 days), and the median procedure time was 60 min (IQR, 45.8–74.8 min).

**Conclusion:**

The ipsilateral pull-through technique is a useful method to detach adhered catheters during difficult TIVAD removal.

**Level of evidence:**

Level 3, Retrospective Study.

## Introduction

Removal of totally implantable venous access devices (TIVADs) can occasionally be challenging. A recent study reported a 7.4% incidence of difficult removals [1], and several endovascular techniques have been developed to address the extraction of adherent indwelling catheters.

Techniques for difficult TIVAD removal are classified into four main ones [2–7]. In the push-in technique, a guidewire is used to straighten the indwelling catheter, followed by a push-in maneuver to detach it from the vessel wall [2]. The sheath-twist technique involves advancing an introducer sheath over an indwelling catheter and then twisting it to dissect the adhesion [3]. In the contralateral pull-through technique, a guidewire is passed through an indwelling catheter and retrieved from the contralateral vein. The adhesion can be released by applying alternating push and pull forces [4, 5]. Lastly, the endoluminal dilation technique uses a balloon catheter advanced through an indwelling catheter; balloon inflation within the catheter releases an adhesion from the vessel wall [6, 7].

However, each technique has certain limitations. For example, the sheath-twist technique cannot be used if the indwelling catheter is too short to accommodate the introducer. An appropriate loop-snare catheter may not be available at some institutions for the pull-through technique. More importantly, the contralateral approach restricts the application of force in the directions parallel to the vessel wall, which can hinder successful removal in difficult cases. We modified the standard foreign-body retrieval technique by using a loop-snare catheter and an ipsilateral approach. This study aimed to demonstrate the difficult removal of port catheters using the ipsilateral pull-through technique.

## Material and methods

### Study design and population

Between December 2015 and April 2025, a total of 3347 TIVAD placement procedures were performed using right/left subclavian vein access (1376/1947 cases), right/left internal jugular vein access (6/4 cases), or right/left upper arm access (9/5 cases). In the same period, a total of 367 TIVAD removal were performed. Among these cases, this retrospective study included patients who underwent TIVAD removal using the ipsilateral pull-through technique. Written informed consent was obtained, and institutional review board approval was secured. Patient demographics (age, sex, etiology, indwelling duration) and procedural data (technique used, procedure time, technical success) were collected. Procedure time was defined from local anesthesia administration to skin closure.

### TIVAD placement

TIVADs were routinely implanted via the subclavian vein under local anesthesia by interventional radiologists. After ultrasound-guided puncture using a long-axis, in-plane approach with an 18-gauge needle (Surflo®; Terumo), a 0.035-inch guidewire (80 cm, Surf; Piolax Medical Devices, Kanagawa, Japan) was advanced under fluoroscopic guidance. A subcutaneous pocket was created, and a 6-Fr port catheter (Anthron P-U catheter; Toray Medical; Tokyo, Japan) was inserted through a peel-away sheath (length, 140 mm; T-handle; Create Medic Co., Ltd., Kanagawa, Japan) with the catheter tip positioned below the carina. The reservoir was then connected, and the skin was closed.

### Standard catheter removal and push-in techniques

Interventional radiologists first attempted standard removal by dissecting the port and catheter from surrounding tissue and applying gentle traction. If resistance occurred, a 0.035-inch guidewire (Radifocus®; Terumo, Tokyo, Japan) was inserted through the catheter and the push-in technique was performed. When unsuccessful, the ipsilateral pull-through technique was used.

### Ipsilateral pull-through technique

**Fig. 1:**
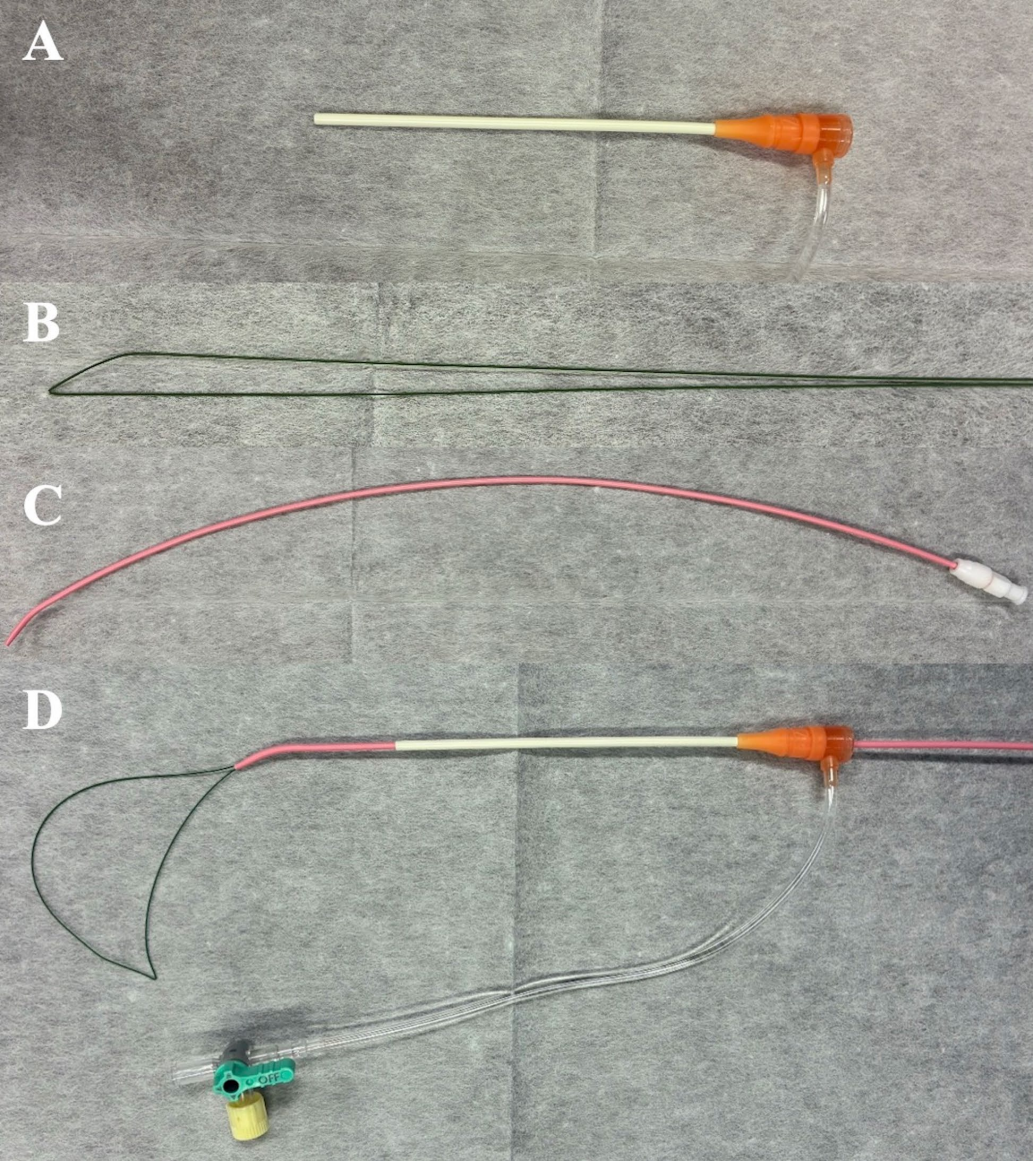
Devices used to create a handmade loop-snare catheter: **A** a 7-French (Fr), 11-cm introducer sheath, **B** 0.018-inch J guidewire folded in half (stainless steel), and **C** 6.5-Fr seeking catheter. **D** The assembled handmade loop-snare catheter

**Fig. 2:**
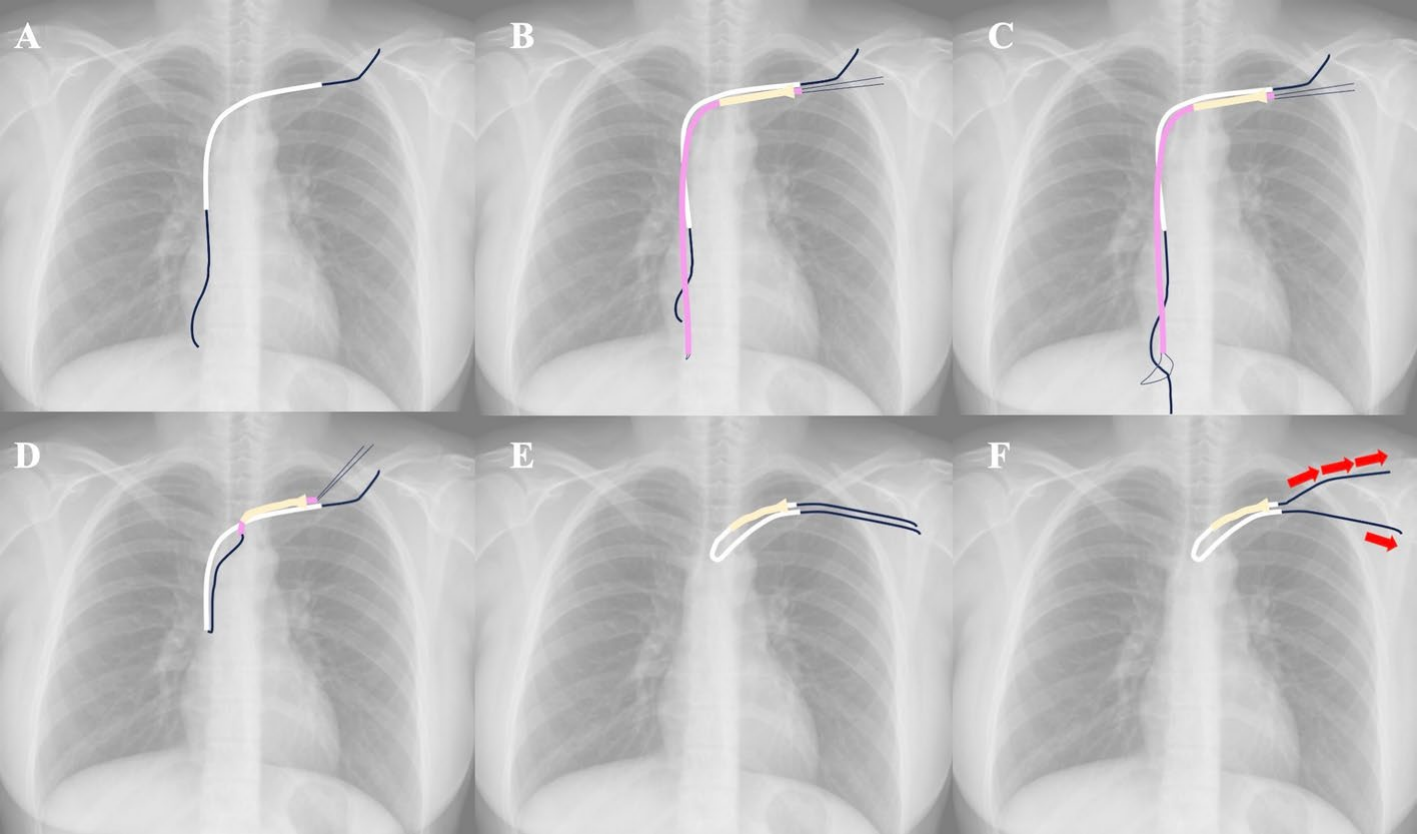
**A** Using pean forceps, the connective tissue around the port was dissected, and the connector between the catheter and the port was removed. A 0.035-inch guidewire (Radifocus Guidewire M; Terumo, Tokyo, Japan) is then advanced through an indwelling catheter. **B** The loop-snare catheter is introduced via the sheath, which was punctured under ultrasound or fluoroscopic guidance, and **C** the guidewire is captured by the snare. **D** The captured guidewire is subsequently retracted into the sheath, and **E** ipsilateral pull-through is established. **F** Pull maneuvers are performed on the indwelling catheter-side and snare-side guidewires. The snare-side guidewire is further retracted, whereas the indwelling catheter-side guidewire is kept in place (arrows in **F** indicate the direction and magnitude of traction). The port catheter was pulled out together with the sheath

The ipsilateral subclavian vein was punctured using ultrasound guidance, or fluoroscopy if ultrasound was unsuitable, and a 7-Fr introducer sheath was placed (Fig. 1A). A 0.018-inch J guidewire (150 cm, Fixed Core type; Argon Medical Devices, Inc., Athens, TX, USA) was folded in half to form a U-loop (Fig. 1B) and advanced through a 6.5-Fr hockey-type seeking catheter (35 cm; inner diameter: 1.24 mm; Hanako Medical, Saitama, Japan; Fig. 1C), serving as a snare catheter (Fig. 1D). After advancing the guidewire through the indwelling catheter (Fig. 2A), the snare catheter was introduced via the sheath (Fig. 2B) and used to capture the guidewire (Fig. 2C). The captured guidewire was then retracted into the sheath (Fig. 2D), establishing ipsilateral pull-through (Fig. 2E). Pull maneuvers were performed using both the snare-side and indwelling catheter-side guidewires, with the former primarily retracted (Fig. 2F). This allowed the catheter, which was adhered to the vessel surface, to be safely separated.

## Results

Among the 367 TIVAD removal procedures, 355 (96.7%) catheters were successfully removed using the standard technique. Three (0.8%) were removed using the push-in techniques, and one (0.3%) was removed using the contralateral pull-through combined with the sheath-twist technique. In 8 (2.2%), the ipsilateral pull-through technique was required after failure of the initial approaches. Patient and procedural characteristics are shown in Table 1. The median age was 60.5 years (interquartile range [IQR], 55.5–67.3 years), and the male-to-female ratio was 1:3. All the patients had cancer. Removal indications were as follows: the port was no longer required in 4 of 8 patients (50%), CRBSI was present in 3 of 8 (37.5%), and catheter malfunction occurred in 1 of 8 (12.5%). One patient had the device implanted at another hospital, and data on the implantation date were unavailable. The median indwelling duration was 2473 days (IQR, 2017–3002 days). The median procedure time was 60 min (IQR, 45.8–74.8 min). Successful removal of the adhered port catheters was achieved through the ipsilateral pull-through technique in 7 of 8 patients. In the remaining patient, the indwelling catheter was firmly adhered along the segment from the left innominate vein to the superior vena cava, where antegrade flow was absent and numerous collateral vessels were present. However, after advancing through the occluded segment and attempting catheter removal, the procedure was aborted due to severe pain and concerns about a potential superior vena cava rupture. In one successful case, a catheter fracture was observed on follow-up computed tomography (CT). No other procedure-related complications were noted.

**Table 1:** Patient demographic and procedural characteristics

Case	Age (years)	Sex	Indwelling duration (day)	Diagnosis	Site	Indication for removal	Removal procedure					Retained catheter on follow-up CT for cancers surveillance
							Procedure time (minutes)	Push-in technique	Contralateral pull-through technique	Ipsilateral pull-through technique	Handmade loop-snare catheter use	
1	71	Female	Unknown^a^	Gastric Ca	LS	CRBSI	71	Failure	Not performed	Success	Yes	Not available
2	57	Female	2935	Breast Ca	LS	Malfunction	101	Failure	Failure	Success	Yes	No
3	28	Male	3451	Osteosarcoma	RS	NLCN	86	Failure	Not performed	Success	Yes	Yes
4	81	Female	3069	Colon Ca	LS	NLCN	27	Not recorded	Not performed	Success	Yes	No
5	63	Female	735	Breast Ca	LS	NLCN	60	Failure	Not performed	Success	Yes	No
6	58	Female	2473	Colon Ca	LS	CRBSI	46	Failure	Not performed	Success	No^b^	Not available
7	51	Male	1577	Colon Ca	LS	CRBSI	45	Failure	Not performed	Success	Yes	No
8	66	Female	2457	Colon Ca	LS	NLCN	60	Not recorded	Not performed	Failure	Yes	Not applicable

*CT* computed tomography, *Ca* cancer, *LS* left subclavian vein, *RS* right subclavian vein, *CRBSI* catheter-related bloodstream infection, *NLCN* no longer clinically necessary

^a^The implantation date was unavailable because the device was implanted at another hospital

^b^A 5-French Angled Wire Loop Retriever (loop diameter: 10 mm; length: 50 cm; Cook Medical, Bloomington, IN, USA) was used

### Case presentation (Case 2, Table 1)

**Fig. 3:**
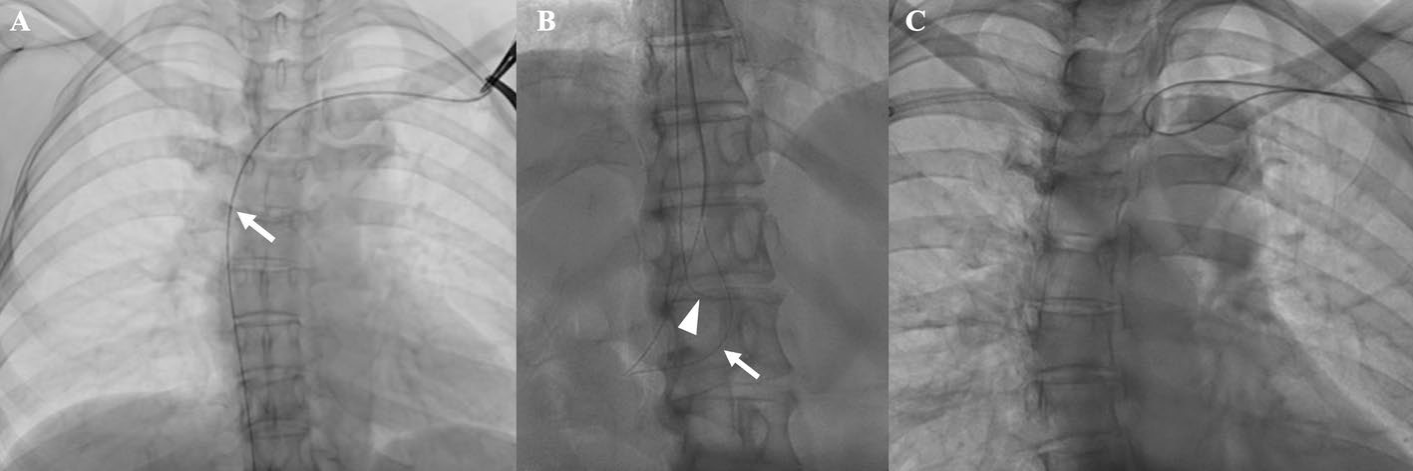
Images of the procedure for removing a totally implantable venous access device via the left subclavian vein in a 57-year-old female with breast cancer. **A** A 0.035-inch guidewire (Radifocus Guidewire M; Terumo, Tokyo, Japan) is advanced through the indwelling catheter into the inferior vena cava. The arrow indicates the tip of the indwelling catheter. **B** A handmade loop-snare catheter is advanced through the ipsilateral subclavian vein using a 7-French introducer. The arrow indicates the loops, which capture the guidewire (arrowhead) through the indwelling catheter in the inferior vena cava. After capture, the snare is tightened against the catheter. **C** The indwelling catheter is withdrawn using the ipsilateral pull-through technique while extracting the 7-Fr sheath. The handmade loop-snare catheter has already been removed

A 57-year-old woman with breast cancer had a TIVAD placed via the left subclavian vein for chemotherapy. After 2935 days of placement, removal of the device and reimplantation via the right subclavian vein were planned due to catheter occlusion. Initially, standard catheter removal and push-in techniques were attempted but met with strong resistance. A contralateral pull-through approach via the right subclavian vein was first attempted but was unsuccessful. Subsequently, the ipsilateral pull-through technique was performed. Under fluoroscopic guidance, the left subclavian vein was punctured, and a handmade loop-snare catheter was introduced through a 7-Fr sheath. The guidewire previously advanced through the indwelling catheter (Fig. 3A) was successfully captured (Fig. 3B), and the catheter was removed without complications (Fig. 3C). Finally, a new TIVAD was implanted via the right subclavian vein. The total procedure time was 101 min, and contrast-enhanced CT performed three months later showed no residual catheter and confirmed patency of the left subclavian and innominate veins, as well as the superior vena cava.

## Discussion

Here, we report successful removal of difficult port catheters through the ipsilateral pull-through technique using a handmade loop-snare catheter formed with a guidewire in 7 of 8 patients. This technique provides improved control and force transmission along the ipsilateral route, thereby increasing the likelihood of successful catheter removal. To the best of our knowledge, although several techniques for difficult TIVAD removal have been described in previous studies, none have detailed this specific approach.

At our institution, TIVADs are routinely implanted via the subclavian vein for several reasons. First, the subclavian approach is associated with a lower rate of catheter-related infection than other sites [8]. Second, it provides better patient comfort than internal jugular access because it causes less foreign-body sensation in their neck [9]. Lastly, the procedure time is shorter with the subclavian approach than with the internal jugular approach, as subcutaneous tunneling is not required [9]. Additionally, the right-sided approach is preferred for specific advantages, including lower risks of lymphatic injury [10] and catheter malposition into the azygos arch [11]. However, the left-sided approach was adopted in cases unsuitable for right-sided access, such as venous occlusion or thrombosis of the right subclavian vein, left pleural effusion, large left lung tumors, or previous catheter placement via the right subclavian vein. An 18-gauge needle was chosen for subclavian vein access to allow single-step placement of a 0.035-inch guidewire, avoiding the two-step upsizing required with a 21-gauge needle. Because an 18-gauge needle may carry a higher bleeding risk than a 21-gauge needle, pre-procedural coagulation testing was performed to assess the patient’s bleeding risk before puncture.

Removal procedures were categorized according to the Wilson grading system as follows: grade 0, no difficulty; grade 1, substantial traction on an indwelling catheter or dissection from surrounding fibrotic tissue; grade 2, endovascular approach; grade 3, venotomy; and grade 4, inability to remove [12]. As surgical approaches, including venotomy, are considered highly invasive, various endovascular approaches have been developed. These approaches have demonstrated high success rates and low risks of complications. Awan et al. reported that the push-in technique using a guidewire was successful in 37 of 42 (88%) cases, with five complications observed, specifically catheter breakage during the procedure [13]. Chan et al. reported that the pull-through technique via the contralateral vein was successful in six of seven (86%) cases, with one procedure-related complication (line embolization, but the fragment was successfully retrieved) [5]. In our study, catheter removal was successful in seven of eight cases, yielding a success rate of 88%, with one minor complication (retained partial catheter). This success rate is comparable to that reported previously.

Although most TIVAD removals are uncomplicated (grade 0 or 1), removal is occasionally difficult. Several studies have identified indwelling duration as a risk factor for difficult removal, reporting a median duration of 1087–1320 days [4, 12, 13]. In contrast, in our study, the median indwelling duration for difficult removal was 2473 days, which was approximately twice that reported previously. Patel et al. suggested a relationship between a longer indwelling duration and increased difficulty in TIVAD removal [4]. TIVAD removal may have been more difficult in our patients than in those reported in previous studies. Nevertheless, our success rates were comparable, suggesting that this technique can be applied to more challenging cases, including those beyond grade 3.

Our technique is unique because it employs an ipsilateral approach for catheter removal. This technique allows the application of force to separate the catheter from the vessel wall. Because this force is directed away from the adhesion site, it may improve the operator’s ability to detach the catheter in more challenging cases. Moreover, it does not require specialized devices or uncommon maneuvers. The diameter of the handmade loop-snare can be easily adjusted in the superior or inferior vena cava, which facilitates the snaring process. The 0.018-inch guidewire we used is made of stainless steel, providing consistent stiffness and strength that facilitates smooth extraction of the 0.035-inch guidewire.

However, our technique has a caveat in that the indwelling catheter may be retrieved in a fragmented state or even break during removal because of forceful stripping from the vessel wall. In practice, we observed catheter fracture in one case, although no major complications occurred afterward. After port catheter removal, the catheter should be inspected for integrity and compared with the documented original length. If a fracture is identified, the fragment must be retrieved immediately, followed by a chest X-ray to detect any remaining catheter fragments. Additionally, our technique may cause increased pain at the catheter-adhesion site during removal; in fact, we terminated the procedure in one case because the patient experienced severe pain, which raised concern for possible superior vena cava rupture.

The limitations of this study include its retrospective, single-center design and small sample size. Furthermore, we were unable to demonstrate the superiority of our removal technique over other methods because several alternative approaches had not been attempted prior to its application.

## Conclusions

The ipsilateral pull-through technique provides an effective salvage option for difficult removal after failure of the standard guidewire technique and represents a practical alternative to existing approaches.

## Data Availability

The datasets generated and/or analyzed during the current study are available from the corresponding author on reasonable request.

## Abbreviations

TIVADs: Totally implantable venous access devices
CRBSI: Catheter-related bloodstream infection
CT: Computed tomography
